# Degradation of dyes using crude extract and a thermostable and pH-stable laccase isolated from *Pleurotus nebrodensis*

**DOI:** 10.1042/BSR20160163

**Published:** 2016-08-05

**Authors:** Xianghe Yuan, Guoting Tian, Yongchang Zhao, Liyan Zhao, Hexiang Wang, Tzi Bun Ng

**Affiliations:** * State Key Laboratory for Agrobiotechnology and Department of Microbiology, China Agricultural University, Beijing 100193, China; † Institute of Biotechnology and Germplasmic Resource, Yunnan Academy of Agricultural Science, Kunming 650223, China; ‡ College of Food Science and Technology, Nanjing Agricultural University, Weigang, Nanjing 210095, China; § School of Biomedical Sciences, Faculty of Medicine, The Chinese University of Hong Kong, Shatin, New Territories, China

**Keywords:** crude extract, decolourization, detoxification, laccase isoenzymes, malachite green, *Pleurotus nebrodensis*

## Abstract

Three laccase isoenzymes (Lac1, Lac2 and Lac3) have been purified to homogeneity from *Pleurotus nebrodensis* in our previous study. Lac2 was shown to be the dominant isoform, capable of oxidizing the majority of laccase substrates and manifesting good thermostability and pH stability. Hence, Lac2 was selected to decolourize structurally different dyes and the colour removal efficiencies of Lac2 and the crude extract of *P. nebrodensis* were compared*.* By monitoring the *λ*_max_ of the reaction system during the course of biotransformation, clear hypsochromic shifts were observed for most of the dyes examined, illustrating that at least one peak disappeared as a result of laccase treatment. In general, Lac2 was more efficient within a short time (1 h) and the crude extract, in general, could achieve similar or even higher efficiency when the duration of treatment was extended to 24 h. Malachite green (MG) was chosen to study the detoxifying potential of Lac2, because of the relatively simple structure and high toxicity of the dye towards microorganisms. The toxicity of MG towards both bacteria (*Bacillus subtilis*, *Bacillus licheniformis*, *Pseudomonas fluorescens* and *Escherichia coli*) and fungi (*Fusarium graminearum* and *Trichoderma harzianum*) was dramatically decreased and the potential mechanism was estimated by GC–MS as to remove four methyl groups firstly and the two newly formed amine groups would be degraded or polymerized further. The present study facilitates an understanding of the application of *P. nebrodensis* laccases and furnishes evidence for the safety of their utilization in the treatment of wastewater emanating from textile industries.

## INTRODUCTION

The textile industry generates huge volumes of wastewater and effluents containing dyes are very recalcitrant to treatment [1]. Conventional treatments are either ineffective or incur considerable costs [2], hence, to develop a technique based on biological treatment seems to be an inspiring solution. By far white-rot fungi are the most efficient microorganisms used for breaking down synthetic dyes [3]. *Phanerochaete chrysosporium*, a well-known lignin-degrading white-rot fungus, has been investigated extensively for its ability to decompose recalcitrant organic pollutants, such as chlorophenols and polycyclic aromatic hydrocarbons [4,5]. However, the key enzyme, peroxidase, for degradation is inhibited by some secondary metabolites secreted by the fungus itself and the degrading ability is evident only in the presence of hydrogen peroxide and veratryl alcohol [5,6]. In contrast, *Trametes versicolour*, another widely studied fungus, releases laccase as the main extracellular enzyme. Laccases can oxidize organic pollutants in the absence of hydrogen peroxide and are not inhibited by secondary metabolites [7]. These copper-containing enzymes catalyse the oxidation of a variety of aromatic compounds, including both phenolic compounds and non-phenolic substrates. Meanwhile, molecular oxygen is reduced to water [8]. Therefore, the application of laccases in the textile industry is an important aspect of bioremediation.

Malachite green (MG), a triphenylmethane dye, has been used as a parasiticide, a fungicide and an antiprotozoan agent because of its wide range of toxicological effects [9]. However, MG has been subjected to controversies due to the risks it poses to human beings; besides, in animals the biodegradation of MG is usually associated with the production of leucomalachite green (LMG), which is colourless but has toxicity [10]. The capacity of various white rot fungal strains to detoxify MG has been verified [11–13].

*Pleurotus nebrodensis*, a delicious and nutritious edible mushroom, is widely cultivated. In our previous work, three novel laccase isoenzymes (Lac1, Lac2 and Lac3) were purified from *P. nebrodensis* and their biochemical properties have been described [14]. In brief, three consecutive ion exchange chromatographic steps on diethylaminoethyl-cellulose (DEAE-cellulose), carboxymethyl-cellulose (CM-cellulose) and quaternary amine-Sepharose (Q-Sepharose) columns and a final gel filtration step on Superdex 75 were employed to yield three monomeric laccase isoenzymes. These laccase isoforms had similar optimum temperatures (55–60°C) and the same optimum pH (pH 3.0). Lac2 manifested the best thermostability and over 70% of its laccase activity remained following exposure to pH 3.0 and to pH 8.0. The laccase isoforms showed great variations in their preferences towards aromatic substrates. Lac2 exhibited the broadest oxidative potential as well as the highest catalytic efficiency towards ABTS (2,2’-azino-bis (3-ethylbenzothiazoline-6-sulfonic acid) diammonium salt), the conventional laccase substrate.

As it is costly to isolate laccases and purified laccases are insufficient for operations on an industrial scale [15], the most feasible way for this application would be incubation of fungi with effluents. Thus, identification of fungal strains that secrete laccases with desirable properties under industrial conditions is a key screening strategy [16]. Hence, the decolourization potentials of the dominant laccase isoform Lac2 and the crude extract were also compared. Meanwhile, the detoxifying capacity of *P. nebrodensis* laccase towards MG was tested as well. The aims of this work were to test the potential utility of *P*. *nebrodensis* water extract and to identify the new laccase isoenzymes as the candidates for industrial applications.

## MATERIALS AND METHODS

### Microorganisms and chemical reagents

The *P. nebrodensis* strain used was obtained from Agricultural Culture Collection of China (ACCC), with ‘ACCC 50867’ as the collection number. Fruit bodies were cultured and purchased from Green Resource (Beijing) Organic Agricultural Technology Development. *Bacillus subtilis* (11088), *Bacillus licheniformis* (10613), *Pseudomonas fluorescens* (10645), *Escherichia coli* (10034), *Fusarium graminearum* (30068) and *Trichoderma harzianum* (32521) were also obtained from Agricultural Culture Collection of China (ACCC). The dyes commonly used in the laboratory (methyl red, bromothymol blue, phenol red, methyl orange, neutral red, basic fuchsin, congo red, erioxchrome black T, coomassie brilliant blue R-250, crystal violet, bromophenol blue, trypan blue, methylene blue, evans blue, MG and remazol brilliant blue R) were purchased from Qiangxin Biorepublic. The dyes commonly used in industries (reactive yellow, reactive brilliant orange K-7R, reactive brilliant orange, reactive red KM-8B, reactive KD-8B, reactive black, reactive navy blue and reactive brilliant blue) were purchased from Jiada Chemical. Laccase substrates were stored in our laboratory. All other chemicals used were of analytical grade unless otherwise stated.

### Assays of *λ*_max_ shifts of various dyes after treatment with crude extract and Lac2

Given the detection limit and resolution of our ultraviolet-visible spectrophotometer, solutions of all tested dyes were adjusted to appropriate concentrations for further studies. A certain amount of dye was first dissolved in McIlvaine buffer (pH 4.0) and a series of dilutions were prepared. Full wavelength scannings (from 190 nm to 800 nm) were conducted to search for the most appropriate concentration for each dye; the maximum absorptions of tested dyes, therefore, were collected. After complete decolourization for 24 h, another set of full wavelength scannings was conducted to record the shifts of *λ*_max_.

### Assays of decolourization potentials of crude extract and Lac2 towards various dyes

Decolourization was measured spectrophotometrically after reaction for 1 h and 24 h at the *λ*_max_ of each dye. Colour removal was tested in the presence of 3 mM ABTS as mediator. The reaction was initiated by mixing 20 μl crude extract or purified laccase (both with 1 unit/ml of laccase activity towards ABTS), 3 μl ABTS and 380 μl dye solution, and the mixture was incubated at 30°C for 1 h and 24 h. For control samples, 20 μl of distilled water was added instead of enzyme and the same conditions were used for incubation. Shift of *λ*_max_ of each dye was then recorded. Decolourizing activity was calculated according to the method of Van Driessel and Christov [17]. All assays were carried out in triplicate.

$$\mathrm{Decolourization}\;\;\left(\%\right)=\left[\left(A_{0}-A_{1}\right)/A_{0}\right]\times 100$$

where *A*_0_ is the initial absorbance of the reaction mixture and *A*_1_ is the absorbance after incubation at *λ*_max_.

### Assays of detoxifying ability of Lac2 towards MG (antibacterial and antifungal experiments)

Firstly, to determine the suitable concentration of MG for each microorganism, a series of MG with concentrations covering 3.125 mg/ml, 6.25 mg/ml, 31.25 mg/ml, 62.5 mg/ml, 156.25 mg/ml, 625 mg/ml and 1250 mg/ml were applied to bacteria and fungi. Inhibition zones were checked after incubation for 12 h for bacteria and incubation for 24 h for fungi. The lowest concentrations leading to inhibition zones with diameters around 1 cm were regarded as suitable concentrations.

The assays of antibacterial and antifungal activities were conducted as detailed by Lam et al. [18]. In the assay of antibacterial activity, sterile petri plates (90 mm × 15 mm) with 15 ml beef extract-peptone medium (2.0% agar) were prepared. Then 5 ml of warm beef extract-peptone medium (0.8% agar) containing bacterium was poured into the plate. A sterile paper disk (0.625 cm in diameter) with 10 μl sterile McIlvaine buffer (pH 4.0) was placed on the agar as control; 10 μl MG solution and 10 μl reaction product were placed on the agar as samples. Plates were incubated at 37°C until inhibition zones were observed. The bacteria tested in the present study included both gram-positive bacteria (*Bacillus subtilis*, *Bacillus licheniformis*) and gram-negative bacteria (*Pseudomonas fluorescens*, *Escherichia coli*).

The assays of antifungal activity towards *Fusarium graminearum* and *Trichoderma harzianum* were performed using sterile petri plates (90 mm × 15 mm) with 15 ml potato dextrose medium. After inoculation of the fungi, plates were left at 26°C for around 48 h to develop colonies. Ten microliters sterile McIlvaine buffer (pH 4.0), 10 μl MG solution and 10 μl reaction product were then placed approximately 0.5 cm away from the rim of the mycelial colony. Plates were incubated at 26°C for 24–36 h until mycelial growth had enveloped disks containing the control and had formed crescents of inhibition around disks containing samples with antifungal activity [18].

### Ultraviolet-visible absorption spectrum monitoring the process of MG degradation

To visualize the bioconversion of MG, the full wavelength scannings (190–800 nm) were continuously monitored from reaction at 0 min to reaction at 2 h, and 10 min was set as the interval between 0 min and 1 h. Scanning result of the reaction at 2 h was obtained right after 1 h. The disappearance and formation of any peaks were carefully recorded and the rate of biotransformation was also analysed to keep a detailed track of this bioconversion progress.

### Analysis of MG biotransformation products by using GC–MS

The MG degradation products were firstly extracted with an equal volume of ethyl acetate three times and the organic layer was collected. Then the organic layer was dried under nitrogen. The dried residue was dissolved in 100 μl ethyl acetate for analytical analysis. GC–MS analysis was carried out using Agilent 19091S-433 interfaced with a mass spectrometer detector. Compounds were separated with an HP-5MS column (30 m long, 0.25 mm id, non-polar). Helium was used as a carrier gas at the flow rate of 1 ml·min^−1^. The injector temperature was maintained at 325°C with the following oven conditions: 100°C kept constant for 1 min, increased to 230°C at a rate of 8°C/min and kept constant for 5 min, and then raised to 280°C at a rate of 10°C/min and kept constant for 25 min. Compounds were identified on the basis of mass value from the mass spectra.

## RESULTS

### Wavelength (*λ*_max_) shifts of dyes after treatment with crude extract and with Lac2

Multiple benzene rings and complicated functional groups led to the high absorbance of dyes at their *λ*_max_. Therefore, a series of dilutions was used to determine the most suitable concentration of each dye for the current study and final concentrations are listed in Table 1. Preliminary study suggested that both crude extract and Lac2 of *P. nebrodensis* would lead to similar spectra after treatment for 24 h (results not shown). The *λ*_max_ shifts monitored during the time course of biotransformation are summarized in Table 1.

**Table 1:** The appropriate concentration required for the decolourization and shifts of *λ*_max_ of the dyes investigated (after treatment for 24 h) *N.C. no change. ^†^N.D. not detected.

No.	Common name	Concentration (mg/l)	Shift in *λ*_max_ (nm)
1	Methyl red	250	524→418
2	Bromothymol blue	125	432→327
3	Phenol red	12.5	431→N.C.*
4	Methyl orange	12.5	460→416
5	Neutral red	12.5	520→N.C.*
6	Basic fuchsin	12.5	530→413
7	Congo red	100	534→494
8	Erioxchrome black T	125	540→750
9	Coomassie brilliant blue R-250	25	549→413
10	Crystal violet	5	584→406
11	Bromophenol blue	25	590→413
12	Trypan blue	12.5	600→528
13	Methylene blue	12.5	698→656
14	Evans blue	12.5	610→541
15	Malachite green	6.25	618→413
16	Remazol brilliant blue R	50	602→N.D.^†^
17	Reactive yellow	100	427→420
18	Reactive brilliant orange K-7R	50	490→393
19	Reactive brilliant orange	50	492→409
20	Reactive red KM-8B	50	520→N.C.*
21	Reactive KD-8B	50	550→514
22	Reactive black	50	585→565
23	Reactive navy blue	25	600→555
24	Reactive brilliant blue	100	605→N.D.^†^

Except for the *λ*_max_ shift of erioxchrome black T, which showed a bathochromic shift (from 540 nm to 750 nm), 18 out of the 24 dyes screened in the present study displayed obvious hypsochromic shifts (*λ*_max_ shifted to a short wavelength), indicating that at least one peak disappeared in each case. Three dyes (phenol red, neutral red and reactive red KM-8B) were resistant to *P*. *nebrodensis* laccases, hence their *λ*_max_ were not changed and no new peaks appeared (N.C. in Table 1). Interestingly, remazol brilliant blue R and reactive brilliant blue seemed to be completely transformed and no peak remained, rendering their absorption curves from 190 nm to 800 nm very flat in appearance (N.D. in Table 1). These results illustrated that dyes were catalytically degraded. Further studies will be needed to identify the products resulting from the decomposition.

### Decolourization of synthetic dyes by crude extract and by Lac2

Decolourization experiments were carried out using a range of dyes, both for laboratory and for industrial usage, including azo dyes (methyl red, erioxchrome black T, etc.), triphenylmethane dyes (coomassie brilliant blue R-250, crystal violet, etc.) and anthraquinone dye (remazol brilliant blue R). Based on the previous study [14], Lac2 was identified as the dominant isoenzyme because of its high thermostability and relatively high pH tolerance. Therefore, Lac2 was selected for degradation study. The degradation ability of the crude extract was monitored simultaneously. Preliminary study also indicated that a higher decolourizing ability towards a broader spectrum of synthetic dyes was obtained in the presence of a redox mediator (ABTS) (results not shown), the following study was carried out in the presence of 3 mM ABTS.

Table 2 illustrates the percentages of decolourization after treatment for 1 h and for 24 h with *P. nebrodensis* laccases. Both crude extract and Lac2 showed efficient decolourizing activity toward six chemically different dyes (erioxchrome black T, coomassie brilliant blue R-250, crystal violet, trypan blue, evans blue and MG). Colour removal was observed after 1 h but higher removal rates could be achieved after 24 h. High efficiencies bringing about over 80% decolourization were observed using coomassie brilliant blue R-250 (84.88% decolourization), trypan blue (86.08% decolourization), evans blue (88.10% decolourization) and MG (96.81% decolourization).

**Table 2:** Percentages of decolourization after treatment for 1 h and 24 h Data are presented as mean±S.D. (*N*=3).

	Colour removal (%)			
	Crude extract		Purified laccase (Lac2)	
Dyes	1 h	24 h	1 h	24 h
Methyl red	0.0±0.02	100±0.04	32±4.0	70.83±4.17
Bromothymol blue	0.0±0.0	31.71±1.63	0.0±0.0	56.46±0.68
Phenol red	0.0±0.0	0.0±0.88	0.0±0.0	20.16±0.81
Methyl orange	0.0±0.0	31.58±2.11	42.42±2.02	68.89±3.33
Neutral red	0.0±0.0	11.58±1.05	12.5±1.04	33.33±2.08
Basic fuchsin	0.0±0.0	40.24±0.0	66.67±3.57	86.90±4.76
Congo red	0.0±0.0	45.0±0.0	15.96±1.06	44.05±1.19
Erioxchrome black T	27.37±0.52	48.20±2.11	42.10±3.16	49.47±1.05
Coomassie brilliant blue R-250	17.5±0.37	83.87±1.61	54.65±2.33	84.88±0.0
Crystal violet	13.54±1.04	60.67±3.37	23.47±1.02	86.73±1.02
Bromophenol blue	0.0±0.11	6.02±2.40	0.0±0.0	14.44±2.22
Trypan blue	57.30±1.12	86.08±0.0	72.04±1.08	81.52±0.0
Methylene blue	16.0±0.10	55.56±3.33	0.0±0.88	0.0±2.75
Evans blue	37.11±1.03	88.10±1.19	78.57±1.02	85.71±1.06
Malachite green	82.69±0.96	96.81±1.06	73.53±0.98	84.54±1.03
Remazol brilliant blue R	0.0±0.0	0.0±2.20	10.64±0.0	84.69±2.04
Reactive yellow	0.0±0.91	0.0±1.25	0.0±0.79	0.0±2.52
Reactive brilliant orange K-7R	0.0±0.6	8.51±1.06	47.0±3.00	89±1.00
Reactive brilliant orange	0.0±0.0	0.0±1.08	4.95±1.98	22.45±1.02
Reactive red KM-8B	0.0±2.36	53.57±2.38	7.29±1.04	48.98±2.04
Reactive KD-8B	0.0±0.92	35.63±3.45	30.11±1.08	56.12±2.04
Reactive black	0.0±0.94	0.0±2.13	0.0±2.88	38.68±1.89
Reactive navy blue	0.0±0.99	0.0±1.32	0.0±3.45	58.76±3.09
Reactive brilliant blue	0.0±0.91	14.74±3.16	13.13±2.02	89.22±2.94

It was found that the crude extract could hardly oxidize some dyes (phenol red, remazol brilliant blue R, reactive brilliant orange, reactive black and reactive navy blue) or could just slightly oxidize other dyes (neutral red, reactive brilliant orange K-7R and reactive brilliant blue) even though the incubation time was extended to 24 h (Table 2). However, colour removal could be achieved once Lac2 was added to these dye solutions (Table 2). The colour removal rates were extremely high for three of the abovementioned dyes, attaining 84.69%, 89.00% and 89.22% for remazol brilliant blue R, reactive brilliant orange K-7R and reactive brilliant blue respectively (Table 2).

Around 35% degradation of methyl orange, basic fuchsin and reactive red KD-8B could be achieved by the crude extract after treatment for 24 h (Table 2), however, once Lac2 was employed, similar decolourizing efficiencies were achieved only after 1 h (Table 2) and treatment for 24 h improved the efficiencies by 20.49%, 37.31% and 46.66% for reactive red KD-8B, methyl orange and basic fuchsin respectively (Table 2).

Methyl red could be effectively oxidized by both crude extract and Lac2 though the crude extract was slow in its action. Similar phenomena were found for congo red and reactive red KM-8B (Table 2). Colour removal by the crude extract was undetectable within 1 h but similar results as those of Lac2 could be observed after treatment for 24 h. After treatments for 1 h and 24 h, colour removal rates for congo red were 45.0% and 44.05% respectively, and colour removal rates for reactive red KM-8B were 53.57% and 48.98% respectively (Table 2).

### Detoxification of MG by Lac2 and proposed mechanism involved

To gain an insight into the detoxifying ability of *P. nebrodensis* laccases and the possible mechanism, several structurally simple dyes, methyl red, methyl orange, basic fuchsin and MG were chosen. Preliminary tests indicated that methyl red, methyl orange and basic fuchsin were nontoxic to tested microorganisms even though they were saturated (results not shown), so MG was selected as the target dye for detoxification study.

**Figure 1:**
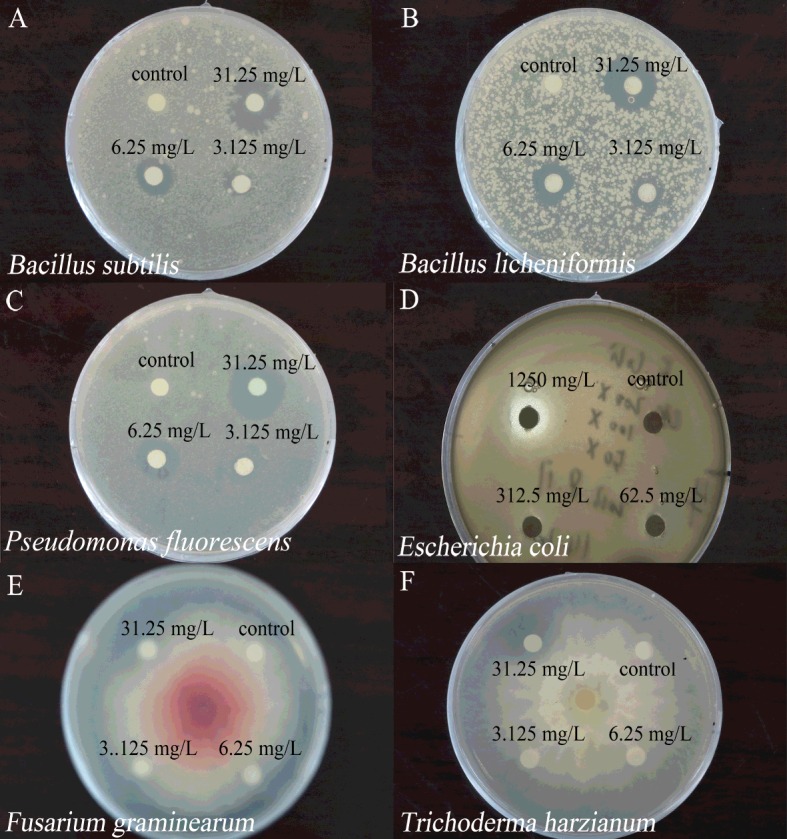
The concentrations of MG required to form appropriate sizes of inhibition zones towards microorganisms (**A**) *Bacillus subtilis*, (**B**) *Bacillus licheniformis*, (**C**) *Pseudomonas fluorescens*, (**D**) *Escherichia coli*, (**E**) *Fusarium graminearum*, (**F**) *Trichoderma harzianum*. Control: McIlvaine buffer (pH 4.0); different concentrations of MG applied to microorganisms are indicated in the pictures.

**Figure 2:**
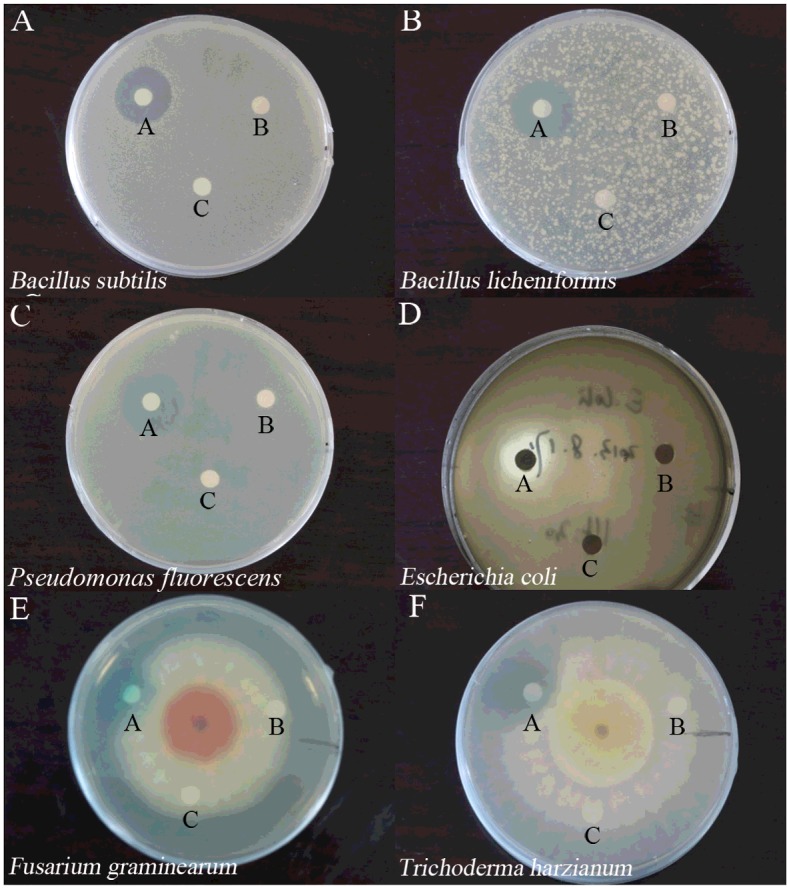
Detoxifying effects of *P. nebrodensis* Lac2 towards MG (**A**) *Bacillus subtilis*, (**B**) *Bacillus licheniformis*, (**C**) *Pseudomonas fluorescens*, (**D**) *Escherichia coli*, (**E**) *Fusarium graminearum*, (**F**) *Trichoderma harzianum*. (**A**) MG (For microorganism (**A**) to (**F**), 31.25 mg/mL, 31.25 mg/mL, 31.25 mg/mL, 1250 mg/mL, 156.25 mg/mL and 62.5 mg/mL MG were applied in sequence); (**B**) control (McIlvaine buffer, pH 4.0); (**C**) products after treatment by Lac2 for 24 h.

The effect of very low concentrations of MG on microorganisms was negligible, whereas no colony was able to recover at very high concentrations of MG. Therefore, after applying a series of concentrations of MG on the tested strains, 31.25 mg/ml, 31.25 mg/ml, 31.25 mg/ml, 1250 mg/ml, 156.25 mg/ml and 62.5 mg/ml MG were regarded as appropriate concentrations for *Bacillus subtilis*, *Bacillus licheniformis*, *Pseudomonas fluorescens*, *Escherichia coli*, *Fusarium graminearum* and *Trichoderma harzianum* respectively (Figure 1). Appropriate sizes of inhibition zones were formed after treatments of tested strains with the abovementioned concentrations of MG (Figure 1). In contrast with the high toxicity of MG (disks labelled ‘A’ in Figure 2), sterile McIlvaine buffer showed no toxicity to tested microorganisms as displayed in Figure 2 (disks labelled ‘B’). Detoxification of MG by Lac2 was proved to be complete since reaction products obtained after 24 h visually exhibited no inhibition on either bacteria or fungi (disks labelled ‘C’ in Figure 2).

**Figure 3:**
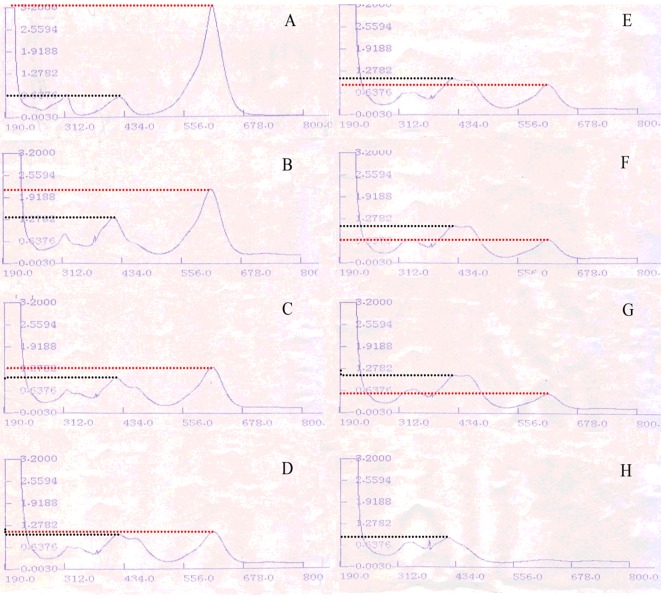
Ultraviolet–absorbance spectra of MG degradation process Ultraviolet–absorbance spectra after reaction for (**A**) 0 min, (**B**) 10 min, (**C**) 20 min, (**D**) 30 min, (**E**) 40 min, (**F**) 50 min, (**G**) 60 min, (**H**) 120 min. Black dashed lines indicate the shifts of absorbance of peak with *λ*_max_ at 413 nm and red dashed lines indicate the shifts of absorbance of peak with *λ*_max_ at 618 nm.

The ultraviolet-visible absorption spectrum of MG was characterized by *λ*_max_ at 618 nm (Figure 3A), and its oxidation product showed *λ*_max_ at 413 nm (Figure 3H). Clear hypsochromic shifts were observed during the biotransformation as recorded in Figures 3(A)–3(H). MG solution, without laccase treatment, displayed three main absorption peaks with *λ*_max_ at 312 nm, 413 nm and 618 nm respectively (Figure 3A). After monitoring absorption spectra within 2 h, the height of the peak at 312 nm remained stable (Figures 3A–3H). The height of peak at 413 nm was doubled within the first 10 min of the reaction and was stabilized afterwards despite the fact that oxidation reaction was uninterrupted, which was marked by black dashed lines in Figures 3(A)–3(H). In contrast with these two less dramatically changed peaks, the peak with *λ*_max_ at 618 nm was completely removed after exposure to the catalytic action of Lac2 for 2 h (Figure 3H), though the bioconversion was slowed down after 30 min (Figures 3D–3H)*.* The red dashed lines in Figures 3(A)–3(H) illustrated the absorption of the peak at 618 nm and the slow-down process was therefore better explained (Figures 3D–3H).

**Figure 4:**
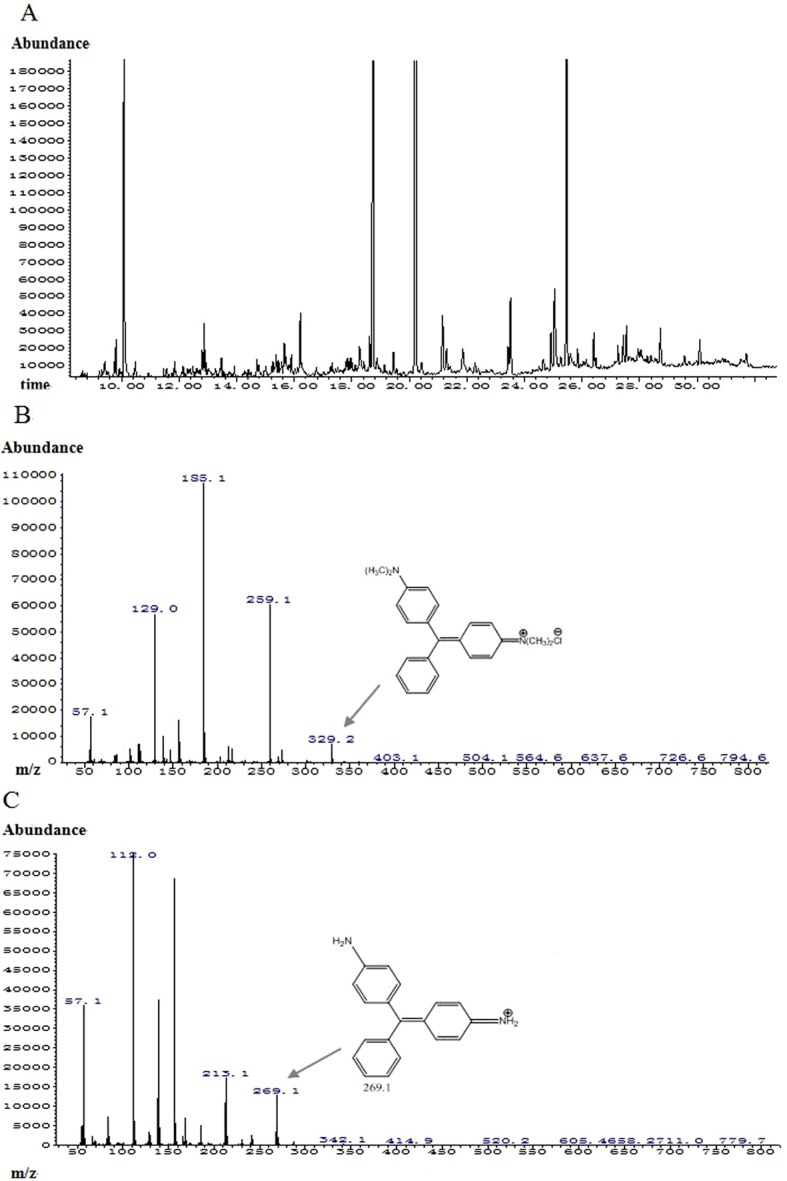
Proposed mechanism of MG degradation by Lac2 (**A**) Total ion chromatography, (**B**) mass spectrum of peak eluted at 20.212 min, mass value of 329.1 represents the molecular mass of MG molecule. Mass values other than *m*/*z* 329.1 are fragments derived from MG, (**C**) mass spectrum at 18.735 min, mass value of 269.1 represents the molecular mass of MG molecule after removing four methyl groups. Mass values other than *m*/*z* 269.1 are fragments derived from demethylated MG molecule.

GC–MS was employed to deduce the degradation products of MG. The total ion chromatogram displayed four major peaks (Figure 4A). After integrating the peak eluted at 20.212 min, the largest fraction exhibited a mass of *m*/*z* 329.1, corresponding to the accurate mass of MG molecule (Figure 4B). By integrating the peak eluted at 18.735 min, the mass value of the largest fraction was *m*/*z* 269.1, corresponding to demethylated MG without four methyl groups (Figure 4C). Unfortunately, based on our current knowledge, no conclusions could be drawn after integrating the other two major peaks eluted at 10.08 min and 25.47 min (results not shown). However, these results were sufficient to suggest the demethylation did occur in the presence of Lac2. The demethylation reaction resulted in the formation of two amine groups, which could be readily oxidized in the presence of laccase and further degradation and polymerization would occur.

## DISCUSSION

It has been documented that laccases from white-rot fungi play a key role in the effluent decolourization process [19,20]. Some *Pleurotus* laccases have also been reported to have colour removal potential [19–21]. However, to date, there has been no report on the application of *P*. *nebrodensis* laccases in any decolouration or detoxification studies. Therefore, an attempt was made in the present study to explore the potentials of *P. nebrodensis*.

Results indicate that both the crude extract of *P*. *nebrodensis* and Lac2 purified from the mushroom have tremendous potential to decolourize different types of dyes. To study the decolourizing ability of dyes belonging to multiple classes instead of only using a limited number as Sathishkumar et al. [22] did is more convincing for discussion about the potential applications of laccases. Ben Younes et al. [23] also made comparisons between one laccase isoform and a crude enzyme preparation from *Perenniporia tephropora* regarding decolourizing ability, but less encouraging results were obtained. *P. nebrodensis* laccases, to some extent, possessed a better potential*.* Though Lac2 was more efficient in colour removal, the crude extract of *P. nebrodensis* was also able to decolourize a great range of dyes when the incubation time was prolonged, suggesting a potential role for *P. nebrodensis* in the remediation of dye-containing industrial effluents.

As mentioned before [24], the oxidation reactions of ABTS and phenolic compounds obey different rules, so even though the crude extract and purified laccases show similar activity towards ABTS, it is very likely they exhibit different effects on dyes. The structures of dyes also play a vital role in efficiency of colour removal. Effects of the different substitutions in the benzene rings of the dye molecules on laccase activity have been reported previously [25]. *N*-Methyl and hydroxyl group substitutions on the benzene ring might be favourable for laccases, and observations of methyl red and eriochrome black T gave full explanations (Table 2); other electronegative substituents of benzene ring like chloro or fluoro did not significantly promote laccase oxidation, which was supported by our observation on bromophenol blue (Table 2).

For most cases, Lac2 displayed better decolourizing capacity than the crude extract (Table 2); however, for the comparative study of the decolourizing efficiency on methylene blue, the crude extract began to remove colour within an hour, but colour removal by Lac2 was indiscernible even when the duration of incubation was lengthened to 24 h, indicating that besides dye structures, the decolourizing performance was dependent on the status of enzymes as well. Gomare and Govindwar [26] reported that the metabolites of dyes can induce or inhibit biotransformation, so the metabolites generated during the degradation process of methylene might cause different side effects on enzymes: Lac2 was more resistant to various dyes and their metabolites, but methylene blue might be more toxic to Lac2 than to other isoforms. The other reason might be that the active sites of Lac2 were more suitable for most dyes than those from other laccase isoforms, but it was not suitable for methylene blue at all. On the other hand, Lac1, Lac3, together with other enzymes from the crude extract, such as peroxidases [27,28], bilirubin oxidase [29] and cellobiose dehydrogenase (CDH) [30], contributed together to colour removal, leading to higher removal rates for methyl red, methyl blue, MG and reactive red KM-8B.

MG is highly toxic and it inhibits the growth of both bacteria and fungi [9], and the observations based on results of Figure 1 lend credence to this conclusion. In the current study, we tested simultaneously the growth inhibiting activity of laccase-treated and untreated MG against microorganisms. The MG-detoxifying ability of *P. nebrodensis* Lac2 was verified by employing both bacteria (*Bacillus subtilis*, *Bacillus licheniformis*, *Pseudomonas fluorescens* and *Escherichia coli*) and fungi (*Fusarium graminearum* and *Trichoderma harzianum*) (Figure 2). The detoxification assays revealed that the reduced toxicity of MG is directly associated with colour removal. The microbial degradation of triphenylmethane dyes by bacteria, actinomycetes, yeasts and fungi has been reported [31]. So far two degradation methods have been found: Yatome et al. [32,33] studied the degradation of crystal violet by *Bacillus subtilis* and *Nocardia coralline* using GC–MS, and the major product was 4,4’ *bis* dimethylamino benzophenone (Michler's Ketone MK), compared with the direct oxidation process, more reports were about the sequential demethylation of triphenylmethane dyes by utilizing HPLC–ESI-MS–MS, such as studies on crystal violet and MG. In the current study, the two major peaks stand for MG [retention time (RT)=20.220 min] and the product after loss of four methyl groups (-CH_3_) (RT=18.735 min). The tiny peaks eluted between 18.735 min and 20.220 min showed negligible intensities (Figure 4). The relatively higher concentration of MG with loss of four methyl groups than those with loss of one, two or three methyl groups is consistent with the findings of Murugesan et al. [12], but the lack of evidence for sequential demethylation indicates a loss of more than one methyl group is possible. Taking into consideration the stability and ability of *P. nebrodensis* laccases to catalyse a wide range of phenolic compounds, the decolourization and detoxification assays indicate that *P. nebrodensis* might be exploited in large-scale waste treatment processes.

## CONCLUSION

Three monomeric laccase isoenzymes were isolated from fruit bodies of *P. nebrodensis.* Lac2 is the dominant laccase isoform and is thermostable and pH-stable. Lac2 more efficiently degraded most of the tested dyes than the crude extract did, but the crude extract could bring about a similar outcome when the incubation time was prolonged. MG toxicity was efficiently removed by Lac2. The process of MG biotransformation was recorded by absorption spectrum and the disappearance of the most dominant peak was consistent with the colour removal rate of MG. The high capacities of colour removal and detoxification of structurally different dyes by *P. nebrodensis* laccases make this fungus a promising candidate in bioremediation of effluents from the textile industry.

## Abbreviations

ABTS: 2,2’-azino-bis (3-ethylbenzothiazoline-6-sulfonic acid) diammonium salt
LMG: leucomalachite green
MG: malachite green
ACCC: agricultural culture collection of China
RT: retention time
